# Well-being and enhancement: reassessing the welfarist account

**DOI:** 10.1007/s11019-024-10246-3

**Published:** 2025-01-10

**Authors:** Anna Hirsch

**Affiliations:** Institute of Ethics, History and Theory of Medicine, LMU Munich, Germany

**Keywords:** Enhancement, Well-being, Welfarist account, Biomedicine, Ethics of enhancement

## Abstract

There are an increasing number of ways to enhance human abilities, characteristics, and performance. In recent years, the ethical debate on enhancement has focused mainly on the ethical evaluation of new enhancement technologies. Yet, the search for an adequate and shared understanding of enhancement has always remained an important part of the debate. It was initially undertaken with the intention of defining the ethical boundaries of enhancement, often by attempting to distinguish enhancements from medical treatments. One of the more recent approaches comes from Julian Savulescu, Anders Sandberg, and Guy Kahane. With their welfarist account, they define enhancement in terms of its contribution to individual well-being: as any state of a person that increases the chances of living a good life in the given set of circumstances. The account aims to contribute both to a shared and clear understanding of enhancement and to answering the question of whether we should enhance in certain ways or not. I will argue that it cannot live up to either claim, in particular because of its inherent normativity and its failure to adequately define well-being. Nevertheless, it can make a valuable contribution to an ethics of enhancement. As I will show, the welfarist account refocuses the debate on a central value in health care: well-being, which can be a relevant aspect in assessing the permissibility of biomedical interventions – especially against the background of new bioethical challenges. To fulfil this function, however, a more differentiated understanding of well-being is needed.

## Introduction

From the beginning, the ethical debate on human enhancement^1^ has been accompanied by the question of an adequate definition of enhancement. As early as 1998, Erik Parens reported that some participants in the debate felt that “the term enhancement is so freighted with erroneous assumptions and so ripe for abuse that we ought not even to use it” (Parens 1998, p. 2). “Enhancement” has also been described as a “slippery customer” (Bess 2010, p. 641). Other authors speak of a “hodge-podge of ill-defined, poorly articulated notions of enhancement” (Earp et al. 2014, p. 5). However, the search for a suitable definition of enhancement is still ongoing. There is widespread agreement in the debate that a common understanding of enhancement is useful for discussing the ethical permissibility and both the advantages and disadvantages of altering or improving human characteristics and abilities (by biotechnological means) (Bess 2010; Savulescu et al. 2011; Gyngell and Selgelid 2016).

While many attempts at definition, especially in the early days of the debate, seemed to be concerned with capturing morally problematic aspects of enhancement and with differentiating it from medical therapies, there are now also approaches that describe enhancement as something inherently positive. This includes the “welfarist account of human enhancement”, which was first introduced into the debate by Julian Savulescu, Anders Sandberg, and Guy Kahane (2011) and then taken up by other authors (Zohny 2015; Gyngell and Selgelid 2016, pp. 117–118).^2^ The welfarist account draws our attention to a connection that intuitively seems perfectly clear, the connection between enhancement and well-being. However, not only is a causal link drawn, but the authors *define* enhancement in welfarist terms as “[a]ny state of a person’s biology or psychology which increases the chance of leading a good life in the relevant set of circumstances” (Savulescu et al. 2011, p. 7).

Savulescu et al. share the view that an explicit and shared definition of enhancement is necessary to resolve debates and arrive at sound ethical conclusions within the ethical debate. However, their account does not seem to have achieved this goal either. Although Savulescu, Sandberg, and Kahane are three well-known authors in the enhancement debate, and their account is now more than 10 years old, it has been relatively little discussed. And when their account is taken up, it is rather criticised than praised for contributing to an explicit and adequate definition of enhancement (e.g. Sparrow 2013; Coenen et al. 2011; Beck and Stroop 2015). There is, however, one author who defends the welfarist account in several papers: Hazem Zohny (2015; 2016; 2019) manages to refute some of the arguments against the account. However, as I will show, he does not succeed in presenting convincing arguments in favour of it.

Why has the welfarist account received so little attention? Is the neglect of the account justified, or does it have a rightful place in the enhancement debate? What is the contribution of the welfarist account to an ethics of enhancement?

The main aim of this paper is not to criticise the welfarist account and banish it from the ethical debate on enhancement. Rather, in order to move the debate forward, I will clarify a possible contribution of the account to the ethics of enhancement – also in terms of the specific benefits that it can offer in comparison with other accounts. I argue that its central value lies in providing normative reasons for or against enhancement – not in providing an adequate definition of it. It highlights the link between enhancement and well-being, which can be a crucial factor in the ethical assessment of enhancement methods, e.g. regarding their legitimacy or justifiability – both in individual cases and in general. However, in order to refer to well-being as a normative criterion for the evaluation of enhancement methods, it seems necessary to specify the concept more thoroughly. The concept of well-being has not been given an appropriate level of concern by the proponents of the welfarist account – nor in the ethical debate on enhancement in general. Further, as I will show, the special importance of health for human well-being should be given a place within these considerations.

Before presenting the welfarist account of enhancement (3), I will briefly discuss the current state of the debate on an adequate definition of enhancement. Thereby reasons for introducing the concept into the debate as well as difficulties in defining enhancement will become apparent (2). Both will help to specify requirements for an appropriate definition/account^3^ of enhancement in bioethics (4). Building on this, I will then point out where I see the main difficulties and inconsistencies of the welfarist account, thereby paying special attention to well-being considerations, (5) and conclude by outlining the potential contribution of the welfarist account to the ethical debate on enhancement (6).

## Enhancement: different accounts, their aims, and problems

Over time, various accounts of enhancement have emerged in the general bioethical and more specific medical ethical debate on enhancement. Most of them are based on a common idea of enhancement, which is already reflected in the word itself:^4^ enhancement as interventions that aim to improve human capacities, performance, or characteristics (Juengst and Moseley 2019). In general, enhancement can include any measures to improve human characteristics, appearance, performance, or capabilities – from coffee at work, to training at the gym, to performance-enhancing drugs. However, so-called ‘mild enhancers’ such as coffee and strength training are usually not included in the debate, as they seem less controversial compared to socially less accepted and more invasive biomedical means such as cosmetic surgery, blood doping, or psychopharmaceutical means. The literature on the ethics of enhancement is predominantly concerned with “biomedical enhancement”: interventions that biologically alter the human body and brain by using pharmaceutical, surgical, or genetic techniques (Juengst and Moseley 2019).^5^

From the outset, the debate has been accompanied by the question of the ethical limits of enhancement: Should everything that is possible be done, or are there (moral) reasons to limit the enhancement of human capabilities? Particularly in the ethical debate on enhancement, some authors seem to have taken the attempt to draw boundaries as an occasion to define enhancement – often in connection with the aim of differentiating it from health-related treatments. An analysis of various accounts of enhancement reveals different reasons that explicitly or implicitly underlie the attempts at definition, e.g. the protection of ‘human nature’, the protection of autonomy and authenticity, the limitation of benefit claims (in the health care system) due to limited resources, the justification of special safety precautions in biomedical measures used for new (non-medical) purposes, and the avoidance of exacerbating existing social inequalities (Juengst and Moseley 2019). Some of these reasons have been rightly criticised, especially the argument that human nature needs to be protected from excessive change (Buchanan 2009; Pugh et al. 2016; Hofman 2017). There is a lot of controversy around the term ‘human nature’ (Roughley 2023). According to some authors it is an essential part of humans to constantly seek to improve and develop themselves, now also by biotechnological means (Bostrom 2003; Harris 2007).

Other reasons behind defining enhancement, on the contrary, seem justified and more important than ever in the light of new ethical challenges. For example, in the context of resource scarcity, which is increasingly aggravated by demographic change, overpopulation, and overconsumption, it seems inevitable to limit entitlements to benefits, also in the health care sector. Questions of justice are closely linked to this issue, e.g.: How can we achieve an equitable distribution of health resources, nationally and globally? The health care systems of Western countries consume many resources and emit large quantities of emissions (Jameton and Pierce 2021). This problem will be exacerbated as more and more medical technologies become more accessible, routine, and used by more and more people – which is undoubtedly a good thing for social justice *within* Western societies. At the same time, there are still parts of the world where people do not even have access to basic health care services. This means an increased provision of enhancement technologies – in countries that can afford to offer these additional services – would not only consume more resources, but also worsen global inequity in health care.

As mentioned above, some accounts of enhancement strive to draw a line between enhancement and medical treatment to mark an upper boundary of professional and social obligations (Juengst and Moseley 2019). Attempts to draw the line are made by the health- and disease-relatedness of treatments versus enhancement (Juengst 1998), the (traditional) goals of medicine (Engelhardt 1990; Benditt 2007; Fukuyama 2002), and the distinction between restoring ‘normal’ human functions versus improving functions beyond the species-typical level of humans (Sabin and Daniels 1994).

So far, all attempts to make a clear distinction between enhancement and treatment have been problematic. Some accounts lead to arbitrary or counter-intuitive distinctions between enhancement and treatment, as shown by, among others, Hofmann (2017) and Jon Rueda et al. (2021). A frequently cited example is the administration of growth hormones, which would be considered a treatment for a boy who is small due to a hormonal disorder, as it is related to a diagnosable disease and therefore falls within the traditional medical remit (Daniels 2000; Rueda et al. 2021). In contrast, the administration of growth hormones to a boy who remains short due to a genetic disposition would be considered enhancement. Even if access to enhancement were to be restricted due to a shortage of resources, would it not be arbitrary to offer hormone treatment in the first case and not in the second? After all, the disadvantages for both boys, e.g. being bullied because of shortness, are the same. A further problem with the treatment-enhancement-distinction (TED) is that these accounts are based on concepts that themselves require clarification or have different meanings depending on the context, such as ‘normal’, health, and disease.^6^ Thus, defining enhancement using these terms automatically leads to conceptual vagueness.^7^

Definitions of enhancement that refer to the goals of medicine are also bound to be ambiguous. It is questionable whether there are clear-cut goals of medicine that would explicitly exclude enhancement as a goal. It goes without saying that medicine today covers many areas that go beyond the traditional understanding of medicine as an ‘art of healing’. These range from preventive medicine to new technologies in reproductive medicine. According to a narrow understanding of medicine as healing art dedicated to curing disease and treating biological dysfunction,^8^ these would not be goals of medicine, and therefore not treatments, but enhancements. Even if that were the case, what would be the consequence? Does it follow that therapeutic measures that pursue ‘genuine’ goals of medicine should always be permitted and financed, whereas measures that do not, which we dismiss as mere self-optimisation or fulfilment of personal desires (Erler 2017), should never be permitted and financed? Does the TED provide a clear boundary between what is obligatory and nonobligatory or between what is permissible and impermissible to offer in health care? There are several reasons for rejecting this. Even medical interventions that can be clearly categorised as disease-related cannot always be provided because of scarce resources or high risks (Daniels 2000).

Conversely, there are medical interventions that, depending on the definition, fall into the realm of enhancement, but which are offered and sometimes financed by insurance companies because they contribute to other important values in our society, e.g. justice or autonomy. These include, for example, contraceptives or abortions, which do not cure disease but give women freedom over their bodies. In addition, regardless of the definition of enhancement, some medical interventions will fall into the grey area between disease-related interventions and non-disease-related interventions. Especially regarding mental problems, it can be difficult to determine whether a diagnosable mental illness is present or not, like in the case of depression – there are still no clear biological markers to indicate when clinical depression begins. Furthermore, there are biomedical interventions that, while having a therapeutic and disease-related goal, not only eliminate disease symptoms but also improve the person’s condition beyond the level before the disease/a certain ‘baseline’. For example, laser eye surgery can not only correct myopia, hyperopia, and astigmatism, but also restore patients’ vision to over 100% visual acuity. Such interventions are somewhere between treatment and enhancement (see also the term “therapeutic enhancement”, Jensen 2020, p. 14).

As has been shown, approaches that refer to TED are fraught with both descriptive and normative difficulties. The TED does not offer guidance as to where the boundaries of the medical profession lie, nor can it clearly identify what interventions should or should not be permitted and offered in health care. Nevertheless, the TED has a certain function within the ethical debate on enhancement, which I will discuss in Sect. 6. Either way, the need to limit access to new biomedical interventions for reasons of resource scarcity and social justice remains. In order to decide whether biomedical interventions that go beyond disease prevention and control should be permitted, offered or financed, having normative criteria based on core values in society seems even more important than having a *clear* distinction between treatment and enhancement.

## The welfarist account of enhancement

Savulescu, Sandberg, and Kahane (2011), who introduced the welfarist account of enhancement into the debate, differentiate between “functional enhancement” as “the enhancement of some capacity or power (e.g. vision, intelligence, health)” and “human enhancement” as “the enhancement of a human being’s life”. In their view, the latter form of enhancement is most relevant to the ethical debate on enhancement, and it can best be defined in “welfarist terms”. Whereas functional enhancement is about improving functioning as a member of the species *homo sapiens*, human enhancement is about improving human life: “The improvement is some change in state of the person – biological or psychological – which is good” (Savulescu et al. 2011, p. 7). According to Savulescu et al. (2011, p. 7), the value to be promoted by human enhancement is “the goodness of a person’s life”, defined as the person’s well-being. From this they derive the welfarist definition of enhancement: “Any state of a person’s biology or psychology which increases the chance^9^ of leading a good life in the relevant set of circumstances” (Savulescu et al. 2011, p. 7). Following the welfarist definition, states of a person, which increase “the chance of leading a good life in the relevant set of circumstances” are “enhancing”, “advantageous” or “abilities”, whereas “disadvantageous states” or “disabilities” are: “Any state of a person’s biology or psychology which decreases the chance of leading a good life in the relevant set of circumstances” (Savulescu et al. 2011, p. 7; Kahane and Savulescu 2009).

According to the welfarist account, it is irrelevant to the classification of an intervention as enhancement whether or not it fulfils medical needs or raises human functions above a ‘normal’ level. Any measure that increases a person’s well-being can be considered an enhancement, whether or not it meets medical needs or increases human functions beyond species-normal levels. Even diminishing human function can be considered an enhancement on the welfarist account, provided that it increases the chances of leading a good life in the relevant set of circumstances, e.g. minimising hearing ability in particularly noisy environments (Earp et al. 2014). In the same way, it would also be enhancement if a person’s IQ is increased even though they already have an above-average IQ – as long as the increase has a positive effect on their life. Conversely, it would not be a form of enhancement to subject a person with a below-average IQ to measures that raise their IQ to or above the average level if it does not increase the chances for the person to lead a good life. According to the welfarist account, enhancement is not about changing human abilities, performance, characteristics, or appearance per se, but about the impact these changes have on well-being (Zohny 2015).

As Savulescu et al. (2011) themselves point out, their account is “inherently normative” as the value of well-being is constitutive of enhancement. Nevertheless, the welfarist account does not imply that enhancement should *always* be permitted and undertaken because of its contribution to individual well-being – it allows for other values, such as justice or the well-being of others, to argue against it.^10^

The TED is not relevant for the welfarist account. Nevertheless, the authors point out that most medical treatments enhance people’s well-being and are therefore a form of enhancement, more precisely a “subcategory” of enhancement. Diseases, on the other hand, are, according to their definition, a subcategory of “disabilities” or “disadvantageous states”. As medical treatments have a very high chance of increasing well-being, they will have higher priority than other measures – at least in most cases. However, the welfarist account is compatible with the fact that in some cases non-medical interventions contribute more to human well-being than medical interventions and should thus be prioritised. As an example, the authors state that raising the IQ of many people with an IQ of 70–80 by 10 points would contribute more to overall well-being than raising the IQ of only a few people with an IQ of 60 by 10 points – even if the former is not medical treatment.^11^

The welfarist account of enhancement also differs from other accounts as measures cannot generally be categorised as enhancement. This becomes clear by the wording “in the relevant set of circumstances”, i.e. according to the welfarist account, cosmetic surgery is *only* considered enhancement if it has a high chance of improving a person’s life by contributing to *their* well-being in the light of *their* circumstances. While cosmetic surgery might increase one person’s chances of a good life and is therefore enhancement, this might not be the case for another person due to different circumstances. Cosmetic surgery, therefore, cannot generally be classified as enhancement.

## An adequate account of enhancement?

Because the welfarist account does not aim at drawing a clear line between medical treatment and enhancement it avoids some of the main difficulties associated with TED. But is the welfarist account itself convincing? Does it contribute in any way to the ethics of enhancement?^12^ And does it offer an adequate definition of enhancement? To answer these questions, it may be necessary to step back and ask more generally: What are requirements for an adequate account of enhancement in bioethics?

Savulescu et al. (2011, p. 3) themselves offer two possible candidates. According to the three authors an adequate definition^13^ of enhancement should

1. allow for a “clear and shared understanding” of enhancement within the debate in order to resolve disputes and reach “sound ethical conclusions” and.
2. help “to agree on answers” on the question of “whether we *should* enhance normal human capacities in these ways.”

These are at least the goals that they want to achieve with their own account of enhancement. Having a shared understanding of enhancement, or at least an understanding that the majority within the debate can agree on, is certainly an advantage, as misunderstandings can be avoided. Without prior explanations, you can jump straight into discussing ethically relevant points. Thus, as far as the second requirement is concerned, it is unclear whether it is still the task of a definition or whether a definition should not rather create the basis for it.

An understanding of enhancement in the bioethical context should adequately capture the term against the background of the specific features of this very context. It is precisely when we pursue ethics with the claim that our considerations will be reflected in ‘real life’ (e.g., that they will contribute to the improvement of global justice in health care or the implementation of ethical guidelines in medical practice), that terms need to be defined in such a way that they can be applied in practice. Following Carnap (1959, pp. 12–18), instead of searching for an adequate definition or understanding of enhancement, one could also speak of “explicating” an already known but vague term with regard to a specific context or a specific objective – in this case the bioethical context. Of course, this also means that the understanding should be helpful in dealing with normative challenges and problems within bioethics. However, it is not the understanding of the term itself, but norms, ethical reasons, and values that are relevant for determining whether a particular intervention is ethically legitimate or not.

Following on from the previous discussion of reasons for defining enhancement and of different accounts of enhancement, I would like to suggest another closely related requirement for an adequate account of enhancement:

3) It should enable us to address (newly emerging) ethical challenges related to biomedical treatments and technologies.

I already mentioned one of these challenges: Given the increasing scarcity of resources and inequalities in health care both within societies and globally, how should enhancement be managed? In the coming years, the possibilities of enhancing individuals and humanity as a whole will increase significantly. However, this trend towards technological innovation will be countered by an increasing scarcity of resources. Consequently, it seems more important than ever to have ethical criteria for assessing the justifiability and permissibility of these technologies.

I will now, against the background of these requirements, critically examine the welfarist account (as presented by Savulescu et al. and Zohny).^14^

## Challenging the welfarist account of enhancement

With their welfarist account, Savulescu et al. try to meet the two requirements stated above. However, it is not clear whether they can be reconciled. But even if one considers them in isolation, limitations of the account become apparent. I would like to start with the first requirement, that of providing a clear and shared understanding of enhancement by referring to Zohny’s defence of the account. Zohny presents a slightly modified version of the welfarist account, but agrees with Savulescu et al. on many points.^15^ Unlike Savulescu et al., however, he ascribes a *purely* definitional purpose to the account.

### Does the welfarist account offer a clear and shared understanding of enhancement that is suited for bioethics?

According to Zohny (2015, p. 123): “The welfarist account is a promising approach to *conceptualizing* enhancement so long as we understand it *only as a definition* of what enhancement is, as opposed to an argument for the permissibility of enhancement” [emphasis added]. The welfarist account is not intended to provide an answer to the question of whether certain technologies should be permitted or not, but rather to “reframe” the concept of enhancement in a way that ties it to the concept of well-being. It is meant to steer the debate back to the relevant question regarding enhancement, namely whether certain interventions can or cannot contribute to individual well-being (Zohny 2015, pp. 125–126).

It remains unclear why Zohny makes this restriction in contrast to Savulescu et al. Maybe he wants to avoid the impression that tying enhancement to well-being necessarily implies a positive valuation of it and a corresponding permissibility. In line with this, he states elsewhere that tying enhancement to well-being does not negate the role of other values, such as justice, in the evaluation of enhancements (Zohny 2015, p. 127; Zohny 2019, p. 608). At the same time, he makes the following statement, which appears to contradict his own position: “Similarly, whether we understand enhancements as interventions that are mere excesses that go beyond restoring normal functioning, or as interventions that contribute to well-being, will have significant implications on how we regulate their use” (Zohny 2015, p. 124). Thereby Zohny suggests that defining enhancement in welfarist terms leads to more positive evaluations of it. In other words, the definition does imply a normative statement. At various points in the text, he departs from his premise that the welfarist account serves only to *define* enhancement, but not to provide an answer to the question of whether or not an intervention is permissible. For example, according to Zohny (2015, p. 125), the account allows us to say whether to favour the development or funding of an enhancement method (not restorative of health) over a nominally therapeutic intervention.

It is questionable whether the majority involved in the debate would agree with this inherently normative understanding of enhancement; after all, not everyone in the debate is a welfarist. Moreover, it may be argued that enhancement does not necessarily aim at increasing individual well-being, as the welfarist account suggests, but that it may aim at contributing to the collective good while accepting limitations of individual well-being. Such considerations might play a role in the debate on moral enhancement (Zohny 2019; Crutchfield 2021; Douglas 2011).

Another aspect of the welfarist definition that will not be accepted by everyone in the debate is the equation of “the good life” with “well-being” – without any further explanation (Zohny 2015, p. 124; Savulescu et al. 2011, p. 4). As a closer look into the philosophical debate shows, there are some reasons to differentiate between “the good life” and “well-being” (Haybron 2020). The good life does not necessarily have to be understood as prudential value *for* the individual subject, as is the case with well-being. A life can be a “good life” even if it is not good for the person living it but good for others (for example, if a person is committed to the well-being of others but has to sacrifice their individual well-being for it). Some authors in the debate link the good life to the category of meaning; according to them, a life can only be good if it is also a meaningful life (Wolf 1997; Metz 2023; Rüther and Murders 2016; Kipke 2014). One need not take this position, of course, but when building enhancement on the concept of well-being or the good life one should comment on their relationship – at least when it comes to philosophical-conceptual considerations.

Even more problematic is the fact that neither Zohny nor Savulescu et al. make a clear statement about their understanding of well-being (or the good life) while claiming to offer a clear understanding of enhancement. As a result, their account shares the same problem as accounts that are based on ambiguous concepts such as health and disease: It is itself ambiguous. Following the welfarist account, enhancement increases the chances of living a good life in the sense of contributing to well-being (Savulescu et al. 2011, p. 7). However, there are many different accounts and theories of well-being. Thus, depending on the account of well-being, different things would count as enhancement (Beck and Stroop 2015). Consequently, one could only have a meaningful discussion about enhancement if one could agree on a definition of well-being – which, given years of philosophical debate about well-being, seems rather unrealistic. Moreover, with some definitions of well-being, it would never be possible to say in general or in advance whether an action is enhancement or not, namely whenever well-being is defined subjectively, i.e. when it is made dependent on subjective attitudes, preferences, or features of the individual person. This would make both a clear and shared understanding of enhancement and a general evaluation of enhancement methods very difficult. It might be true that the three main theories of well-being in the philosophical debate (hedonism, objective list theory, and desire-fulfilment-theory)^16^ might often agree on *what* contributes to well-being and what does not (Savulescu et al. 2011; Savulescu and Kahane 2011; Savulescu and Kahane 2009; Zohny 2015) – even though they might not agree on the explanation for it. However, if we assume that well-being depends, at least in part, on subjective attitudes, preferences, or characteristics, then it would not be possible to define enhancement in general terms and without consulting the person who is to be enhanced.

Despite these criticisms, does the welfarist account provide an adequate definition of enhancement *for* the bioethical context? On the one hand, yes, because many biomedical technologies used in medicine serve to increase the well-being of people. The welfarist account thus draws attention to an important aspect in the evaluation of biomedical interventions. On the other hand, understanding the long-established concepts of therapy and medical treatment^17^ as “subcategories” of enhancement will lead to resistance and counterintuitive consequences, especially in the context of patient care. If medical treatments are defined in terms of their contribution to well-being, can an intervention be a medical treatment for one person and not for another, despite the same medical conditions and external circumstances? The answer depends on the underlying understanding of well-being.

On the basis of a purely objective understanding of well-being, which defines well-being in terms of human functioning and ignores the perspective of the well-being subject, this wouldn’t be possible. Only by taking into account subjective aspects of well-being, be they attitudes or other characteristics, can the same intervention be a treatment in one case and not in another – under the same external circumstances. But how does this categorisation help us? In terms of *definition*, the categorisation would probably lead to confusion – if, for example, you were to say to a patient: “This is an intervention I am offering you that could increase your chances of living a good life from a medical point of view, but it is only a ‘medical treatment’ if it also contributes to your subjective well-being.” That would not only be confusing, but also unhelpful.

The TED is undoubtedly worthy of criticism and leads to counterintuitive consequences. But it seems also highly counterintuitive to subsume therapies under the term enhancement.

### Does the welfarist account of enhancement help to answer the question “whether we should enhance in certain ways”?

As with the attempt to define enhancement in welfarist terms, the question arises as to how the second requirement can be met without a sufficiently clear definition of well-being. The problem is illustrated, in the following statement by Zohny (2015, p. 125): “As it happens, things that we think of as therapies tend to contribute more to our well-being than interventions that might improve our functioning beyond some norm. […] Under the welfarist account, this gives us *concrete*,* normative reasons* to prioritise such therapies over such enhancements” [emphasis added].^18^ It remains unclear how the welfarist account is supposed to provide “concrete, normative reasons” without being based on a clear understanding of well-being. Moreover, it would not be the welfarist account itself that provides the normative reasons, but the understanding of well-being on which it is based.

Since Zohny assumes that therapies tend to contribute more to our well-being than non-medical interventions, it seems as if he tacitly assumes an objective understanding of well-being that assigns a high relevance to basic functions (see Sect. 6). Therapies usually serve to eliminate symptoms and restore basic functional capabilities of the human body, while enhancement measures – as they are commonly understood – go beyond it. Elsewhere, however, it seems that Zohny does not presuppose a purely objective understanding of well-being, but one that establishes a relationship to the well-being subject: “What this shows is that there is no context-independent answer to the question of whether a state increases or decreases the chances of leading a good life. The particular circumstances of the individual […] clearly play a determining role in answering that question” (Zohny 2019, p. 607). Referring to Alicia Hall and Valerie Tiberius (2016), Zohny seems to favour an objective theory of well-being which is “subject-relative” or “subject-dependent”. According to them an objective theory of well-being can also be *subject-relative* if it refers in some way to characteristics of the subject – even if, unlike a *subjective* theory of well-being, it does not make well-being dependent on *subjective attitudes*.

Savulescu et al. (2011, p. 16) themselves admit that – on a welfarist account of enhancement – whether we should intervene depends, among other things, on “[t]he account of well-being we employ”. Similar to Zohny, they don’t explicate their understanding of well-being but seem to prefer an objective and at the same time subject-relative theory. At least this understanding is evident in their account of disability: According to Savulescu and Kahane (2009, 2011) disability (defined in welfarist terms as states that are detrimental to well-being, see Sect. 3) is relative to a specific person.^19^ For example, deafness and dwarfism can diminish well-being in certain life circumstances, while in others they can contribute to well-being and are thus – according to their definition – not disabilities.^20^ Parents with dwarfism would claim that they can take better care of their child if they are also dwarf, which would get the child a better life. Nevertheless, Savulescu and Kahane admit that we need “to speak in generalities” in various contexts as “certain foods, substances, temperatures, etc., are harmful to most human beings” (Kahane and Savulescu 2009, p. 27). So, it seems as if Savulescu et al. do have a certain understanding of well-being in mind. However, it remains unclear why they don’t make it explicit.

In summary, there is a lack of clarity about what the welfarist account is supposed to achieve. It has proven problematic to try to establish an inherently normative understanding as a clear and shared understanding of enhancement within the bioethical debate. In addition, it became apparent that the two requirements can only be achieved if more is said about the understanding of well-being on which the account is based. However, regarding the third requirement (*Does the welfarist account of enhancement help to address (emerging) ethical challenges within an ethics of enhancement?*) the welfarist account can indirectly make a valuable contribution that neither Savulescu et al. nor Zohny explicitly point out – which I will make up for in the following section.

## How the welfarist account can contribute to the ethics of enhancement

As shown above, two of the reasons for limiting access to biomedical interventions are particularly relevant due to current ethical challenges in health care: the necessary limitation of entitlements in the health care system due to resource scarcity and demographic change and the exacerbation of existing inequities in health care (nationally and globally). It also became evident that in order to judge whether biomedical measures and innovations should be allowed/developed/financed or not against the background of these pressing ethical challenges, there is no need for a *strict* boundary between measures or innovations that can be clearly assigned to the medical-therapeutic field and those that are commonly regarded as enhancements. It seems much more important to have normative evaluation criteria based on central values in our society, to guide us in our decisions. And this is where the welfarist account comes into play.

As Savulescu et al. (2011, p. 7) rightly point out, one benefit of their account is that it reconnects enhancement with the value of well-being. In contrast, the enhancement debate tends to focus on a different value, the value of autonomy. Discussions are either about how enhancement can promote our autonomy or how enhancement threatens it (Lewis 2021; Bandeira and Lenine 2022; Schaefer et al. 2014; Juth 2011; Heilinger and Crone 2014). The debate on enhancement and autonomy is undoubtedly an important one. Enhancement often falls into areas of wish-fulfilling medicine, such as cosmetic surgery and anti-ageing medicine. In these areas of medicine, patients do not generally seek treatment because of a diagnosable disease, but because of personal desires. However, while the focus of aesthetic surgeons may be on fulfilling patient wishes, they are, of course, still committed to the well-being of their patients in their role as physicians. Whether they offer a patient a particular procedure should therefore depend not only on considerations of autonomy, but *also* on considerations of well-being; on considerations of whether the procedure has a chance of contributing to the patient’s well-being given their personal circumstances. Offering patients procedures that are unlikely to contribute to their well-being is not only detrimental to the individual patient, but also to society as a whole, as resources are wasted for no or the ‘wrong’ reasons. One (rather extreme) example is to offer cosmetic surgery to a patient with body dysmorphic disorder.^21^

The value of autonomy and the resulting obligation to respect patient autonomy are not in themselves helpful when it comes to questions of the permissibility of certain enhancement measures. People have all kinds of desires and claims what medicine should do for them – which are not necessarily compatible with the well-being of others, social justice, or the economic use of resources. When evaluating the permissibility of enhancement methods, therefore, other values need to be considered as well: justice, of course, but also well-being. A purely subjective understanding of well-being, according to which well-being consists of the fulfilment of personal desires, would not do the job either. In this case, the promotion of well-being would coincide with the duty to respect patient autonomy; if health care professionals wanted to benefit patients, they would simply have to fulfil their wishes. Then, however, we would be back to the problem with the exclusive reference to the value of autonomy in assessing enhancements.

What would it mean, then, to incorporate the value of well-being *more fully* into normative considerations of whether to provide certain enhancement measures?

For the further development, approval, and funding of enhancement interventions, it follows that they would have to be tested even more closely for their benefit to the well-being of their potential users. In order to do this at a general level, an objective understanding of well-being seems necessary; for the individual user, it would have to be applied to their personal circumstances and characteristics in the sense of subject-relative well-being. This seems to be in line with the understanding of well-being that Savulescu et al. and Zohny prefer without making it explicit.

How could this idea be further developed?^22^ So, first, to the objective core of well-being: It might consist of basic goods that can be assumed to contribute to the well-being of every human – or at least to the well-being of most people. In addition to well-being goods that affect our health, goods regarding other areas of life (relationships, self-fulfilment, etc.) should also be taken into account, since human well-being is not just about health. And even if medicine focuses on health-related well-being, it too has long transcended it (see Sect. 2). Brock (1993) makes the following distinction regarding quality of life: On the one hand, there are “primary functions” that are valuable in any human life plan – in fact for agency in general. These include goods that are part of human health, such as well-functioning organs, but also non-health-related well-being goods or goods that are only indirectly related to health, like ambulation or communicative skills. On the other hand, there are “agent-specific functions” whose value for well-being only becomes apparent against the background of personal life plans and preferences. They may also be related to health, for example the “physical dexterity needed for success as a musician, surgeon, or athlete” (Brock 1993, p. 127).

At this point, it could be argued that basic goods or primary functions are not helpful in the assessment of enhancement, since enhancement interventions always go beyond these basic functions. As the welfarist account shows, however, it can be questioned whether enhancement is to be understood in this way. Moreover, nominally therapeutic interventions also go beyond the protection and promotion of primary functions (preventive medicine, reproductive medicine, etc.), and, as shown, some medical interventions fall in between, such as restoring a person’s vision with laser eye surgery beyond the ‘baseline’.

However, it seems right that enhancement is more often aimed at promoting well-being goods that fall within Brock’s agent-specific functions. For example, for most people it is sufficient to hear well enough to navigate safely through life, enjoy pleasant sounds like music, and communicate with others. However, a musician might consider it important for their well-being to have absolute hearing. If there were a biomedical intervention that could provide absolute hearing, such as a special hearing aid, this *might* be more beneficial to the musician’s well-being than an intervention that fixes their limited walking mobility and thus restores a primary function. In *individual* cases, therefore, one might conclude that an intervention to promote agent-specific functions would contribute more to well-being than an intervention to promote primary functions.

Brock’s distinction seems to be helpful in determining the benefit of enhancement interventions in individual cases, but also on a general level? It appears that there is an overlap between specific life plans beyond primary functions, so that for some agent-specific functions it can be said that they are relevant in more life plans than in others. For example, if most people within a society consider it relevant to their well-being to have children, then it makes sense to provide better access to new reproductive technologies than to new ways of reducing wrinkles. This is consistent with the fact that medicine always has been influenced by values and interests of society (Juengst 1997; Hofmann 2001; Juth 2011). So, when it comes to the question of how best to use limited resources in health care, well-being seems to be a helpful aspect to consider – both at an individual and societal level.

Regarding the assessment in individual cases, it is also worth highlighting an aspect that Earp et al. 2014 point out as an advantage of the welfarist account. Contrary to what the debate on enhancement sometimes suggests, ‘more’ does not always mean ‘better’ (for example, excellent hearing in noisy environments). Thus, it must always be considered whether more of something is in fact conducive to well-being, or whether a deliberately limited use or even omission of enhancing certain capabilities, characteristics, etc. would have a greater chance of increasing overall well-being – also bearing in mind that the enhancement of individual abilities or certain aspects of appearance may conflict with the protection of primary functions. For example, excessive breast augmentation can lead to back pain, rhinoplasty can affect breathing, and steroid use can cause infertility. It is the responsibility of medicine and society as a whole to consider this when assessing the permissibility of biomedical interventions, not only to protect the general well-being of patients, but also to avoid preventative follow-up procedures. A more differentiated understanding of well-being can be of help here.

Concerning preventable biomedical interventions, existing measures that are offered and performed without much thought, like braces and aligner treatment for mainly cosmetic reasons, should also be questioned – regardless of whether they fall into the area of treatment or enhancement according to common definitions. For years, some procedures have simply been offered without questioning whether they can actually promote the well-being of patients in the long term. Here, too, resources could be saved and negative effects on the environment avoided, e.g. plastic waste from aligner therapies (Veseli et al. 2024). Of course, such considerations are countered by the pressure on clinics and practices to be cost-effective – discussing this would be beyond the scope of this article.

In order to decide on the admissibility and funding of different biomedical interventions when resources are limited, it thus seems necessary – especially when decisions are made at a general level – to have criteria for assessing and balancing the potential benefits of these interventions. Answering the following questions may be helpful here: How *fundamental* to well-being are the goods promoted by different biomedical interventions? Can they *substantially* improve people’s lives (*in different areas*)? Building on this, it is possible to explain (and not just assume) why disease-related interventions often contribute more to well-being than non-disease-related interventions. More fundamental well-being goods, like primary functions, often concern human abilities and characteristics that are related to our health – in a narrow medical sense, but also in a broader, positive sense.

Against this background, it may not be entirely correct to dismiss TED altogether, as proponents of the welfarist account do – even if we cannot draw a strict line between enhancement and therapy. As elaborated, the special significance of well-being goods concerning basic human functions/health shows that TED points to morally relevant categories in the discussion of biomedical interventions. Among other things, it reminds us that a certain minimum level of health is necessary to achieve well-being at all, and that there is a certain qualitative difference between disease-related therapies and enhancement, even if this is not always as clear-cut as some proponents of TED claim (Bess 2010). In many cases, disease-related constraints limit the ability to lead one’s own life more significantly than the absence of certain agent-specific functions (Malmqvist 2014). The professional musician, for example, can be an excellent musician without perfect hearing. But if osteoarthritis prevents them from moving their fingers, they will be limited not only in playing the piano, but also in their everyday life. These considerations argue against abandoning the concept of therapy/treatment and subsuming it under the concept of enhancement.

So, getting finally back to the question posed at the beginning, whether the welfarist account has a rightful place in the enhancement debate, the answer is “yes”. By emphasising well-being, the welfarist account refocuses the debate on an important value both in medicine and in society in general. Regarding the question whether it does contribute to the ethical evaluation of enhancement methods, however, the answer is only a conditional “yes”. In order to make this contribution, the welfarist account needs to be more sophisticated in its understanding of well-being.

## Concluding remarks

In the coming years, there will be more and more opportunities to enhance individuals and humanity as a whole. However, the trend towards technological innovation is being countered by the increasing scarcity of resources, the shortage of health care professionals, the rise in social inequalities, and demographic change (more people are living longer, are fitter, and want to maintain their functional capacities for longer and better). For this reason, it seems more important than ever to have normative standards for assessing whether certain measures should be developed, offered, and financed – even more important than having a shared understanding of enhancement.

In this paper, I examined whether the welfarist account can contribute to dealing with this challenge and to an ethics of enhancement in general. Referring to the discussed requirements for an adequate account of enhancement (Sect. 4), it can be stated that: First, the welfarist account does not provide a clear and shared understanding of enhancement for the bioethical debate. It is inherently normative, and it is questionable whether the majority in the debate would agree with this understanding of enhancement. Second, the welfarist account does not help to agree on answers whether we should enhance in certain ways or not – at least not on its own. And third, taken by itself, it cannot address (emerging) ethical challenges posed by an ethics of enhancement. However, as I have shown, it could meet the second and the third requirement. While drawing attention to an important value in the evaluation of enhancement, well-being, the welfarist account fails to provide a clear and adequate definition of it. A subject-relative understanding of well-being that includes objective elements, e.g. in the sense of Brock’s primary functions or ‘fundamental building blocks’ of human well-being (often health-related), seems to be a good starting point regarding the assessment of biomedical interventions.

This article argued that the concept of well-being should have a more prominent place within an ethics of enhancement, and offered a glimpse of *how* it can enrich the debate on biomedical enhancement. It is now an important task to advance this idea and elaborate an appropriate understanding of well-being in the context of enhancement – only then can well-being function as a helpful criterion for the evaluation of biomedical interventions which aim to improve human capabilities.

## Footnotes

1 To simplify, I will use “enhancement” (instead of “human enhancement”) in the following.

2 Another more recent approach in the enhancement debate, is the “functional augmentative approach of enhancement”, proposed by Nick Bostrom and Rebecca Roache (2007). According to this approach, enhancement is any increase in a person’s capacities or functions – “regardless of how that capacity is distributed statistically” (Martín et al. 2023).

3 “Definition”, “account”, “approach”, and “understanding” are often used interchangeably in the enhancement debate. In general, most authors want to give more than just a descriptive definition of enhancement. So maybe it is more appropriate to speak of “accounts” or “approaches” of enhancement than of “definitions”.

4 According to the *Oxford English Dictionary* “to enhance” means: “To raise in degree, heighten, intensify (qualities, states, powers, etc.)” (https://www.oed.com/search/dictionary/?scope=Entries&q=enhance, accessed 31 May 2024).

5 My focus will also be on biomedical enhancement. When I refer to enhancement below, I therefore mean biomedical enhancement.

6 For a concise summary of the criticisms that have been levelled against TED in recent years, see Rueda et al. 2021.

7 There are, for example, very different ways of understanding health (for an overview see Murphy 2023). If you take a very broad understanding of health, as the World Health Organization (1948) does, many interventions will still fall within the scope of treatments. On the other hand, if we use a very narrow understanding of health, such as Christopher Boorse’s (1975) biostatic model, many of these interventions would be classified as enhancements. Without having a clear understating of health, it is therefore not possible to distinguish treatments from enhancement on the basis of their health-relatedness.

8 Some of these definitions include the prevention of disease and thus preventive medicine, but still narrowly define the scope of medicine. For example, Daniel Callahan (1999, p. 104) identifies four goals that should be at the heart of the work of medical practitioners: The prevention of disease and injury, the relief of pain and suffering caused by disease, the care and cure of the sick, and the care of those who cannot be cured, the prevention of premature death, and the pursuit of a peaceful end to life. Callahan opposes medical interventions that go beyond the maintenance and/or restoration of health because they would alter the human condition too much.

9 The authors note that “expected to increase the chances of leading a good life” is meant probabilistically. Something that is “expected” to increase the chances of leading a good life, does *not necessarily* result in a good life. They take the term “expected” from decision theory: “The expected value of an outcome is the value of that outcome multiplied by the probability of it occurring. In the debate around enhancement, the outcome of value is a person’s life and how well it goes” (Savulescu et al. 2011, p. 9).

10 However, Savulescu and Kahane’s strong pro-enhancement stance and prioritisation of the value of well-being becomes very clear in the debate on genetic enhancement and procreative choice (Levin 2023; Malmqvist 2014). According to the *Principle of Procreative Beneficence* couples, “who decide to have a child have a significant moral reason to select the child who, given his or her genetic endowment, can be expected to enjoy the most well-being” (Savulescu and Kahane 2009, p. 274; cf. Savulescu 2001).

11 They do not further substantiate the connection between a higher IQ and overall well-being at this point. However, they emphasise elsewhere (Savulescu et al. 2011, p. 13) that people with an average IQ are generally better placed to participate fully in social life in a technologically advanced society. In contrast, people with a below-average IQ are excluded from important options within societies, e.g. employment opportunities. Elsewhere (p. 11) they point out that although intelligence does not automatically make a person happier, empirical studies indicate that it can protect against mental and health problems and thus contribute to overall well-being.

12 In fact, Brian D. Earp, Sandberg, Kahane, and Savulescu (2014, p. 4) themselves raise questions about the usefulness of the welfarist account: “Well… so what? What does this welfarist definition get us? How will it be useful for medical professionals, neuroethicists, and other stakeholders engaged in these sorts of discussions? Finally, what advantages does it have over other definitions used throughout the literature?” The only reason they give, is that the welfarist account makes it clear that ‘more’ does not always mean ‘better’ (see Sect. 6).

13 Savulescu et al. (2011) also do not differentiate between an “account of”, a “definition of”, and an “approach of” enhancement.

14 The most common criticisms have been summarised and – in some cases – convincingly refuted by Zohny (2015, 2019).

15 Zohny, Savulescu, and Earp recently published a joint article on the welfarist account (Zohny et al. 2022), suggesting that they are largely in agreement. Thus, in what follows I will mainly speak of the welfarist account in general. However, where I am responding only to statements by Savulescu et al. *or* to statements by Zohny, I will make it clear. I am not referring to the modified version of the welfarist account proposed by Chris Gyngell and Michael J. Selgelid (2016), which is compatible with TED (Gyngell and Selgelid 2016, pp. 117–118).

16 This classification was introduced by Derek Parfit in *Reasons and Persons* (Parfit 1986, p. 493).

17 “(Medical) treatment” and “therapy” are often used interchangeably in the literature. Strictly speaking, however, they have different meanings. “Therapy” is a specific type of treatment “that helps someone feel better, get stronger, etc., especially after an illness” (https://dictionary.cambridge.org/dictionary/english/therapy, accessed 19 August 2024). Referring to medical care, “treatment” is defined as “the use of drugs, exercises, etc. to cure a person of an illness or injury” (https://dictionary.cambridge.org/dictionary/english/treatment, accessed 19 August 2024).

18 He compares “mending a broken limb or treating cancer” (therapies) with “augmenting normal eye sight or concentration levels” (Zohny 2015, p. 125).

19 For a critical analysis of the welfarist account in the context of procreative decisions and disability, see Levin 2023. Among other things, Susan B. Levin points to the inadequate engagement of the welfarist account with the concept of well-being within this context.

20 For a critique of this revisionist understanding of disability, see, for example, Campell and Wasserman 2020.

21 Persons with body dysmorphic disorder (BDD) are constantly preoccupied with (sometimes only minor or supposed) ‘defects’ in their appearance – often to such an extent that they are restricted in their social, affective or other behaviours. Cosmetic surgery does not usually lead to greater satisfaction in these patients. On the contrary, it creates a desire for further operations, resulting in a chronic situation (Vindigni et al. 2002).

22 Jon Rueda et al. (2021) suggest using the capability approach to assess enhancement interventions.
